# Case Report: Dislocation of lateral menisci secondary to congenital lateral tibiofemoral cartilage thickening in both knees

**DOI:** 10.3389/fsurg.2024.1395276

**Published:** 2024-07-12

**Authors:** Xingliang Zhang, Wentao Li, Zijin Li, Yibing Xie, Chenyu Zhu, Shaoyang Lei, Shuqian Zhang

**Affiliations:** ^1^ Department of Graduate School, Hebei Medical University, Shijiazhuang, Hebei, China; ^2^ Department of Radiology, Hebei General Hospital, Shijiazhuang, Hebei, China; ^3^ Department of Radiology, The First Teaching Hospital of Tianjin University of Traditional Chinese Medicine, Tianjin, China; ^4^ Department of Radiology, Yuebei People’s Hospital, Shaoguan, Guangdong, China; ^5^ Department of Graduate School, Hebei North University, Zhangjiakou, Hebei, China; ^6^ Department of Graduate School, North China University of Science and Technology, Tangshan, Hebei, China

**Keywords:** cartilage thickness, functional adaptation, magnetic resonance imaging, knee joint, spectral CT

## Abstract

A 24-year-old male patient complained of mild knee pain after jogging. The subsequent knee MRI demonstrated bilateral lateral thickened tibiofemoral cartilages, evidenced by deformities of the bilateral subchondral bone beneath the lateral femoral condyle cartilage. The corresponding dislocations of almost all the left lateral meniscus and part of the right lateral meniscus to the center of the joint were detected. After excluding diagnoses of congenital ring-shaped meniscus, bucket handle tear of the C-shaped lateral meniscus, and central tear of the discoid meniscus, the displacement of all or part of the lateral meniscus into the intercondylar notch was considered a consequence of congenital thickening of the lateral superior and inferior cartilage. This case may report a new variant of knee joint pathology.

## Introduction

Congenital thickening of the articular cartilage of the knee joint has rarely been reported previously (1). Herein, we report an extremely uncommon adult case, based on MRI, involving bilateral-involved lateral meniscus variants, which manifested as the displacement of the meniscal structure to the intercondylar notch (2), complicated by bilateral-involved congenital thickening of cartilages covering both the lateral femoral condyle and tibial plateau. Key points of differential diagnosis from ring-shaped meniscus (RSM) and bucket handle tear (BHT) were discussed. A new variant of the lateral meniscus secondary to the thickened superior and inferior articular cartilages was addressed.

## Case description

**Figure 1:**
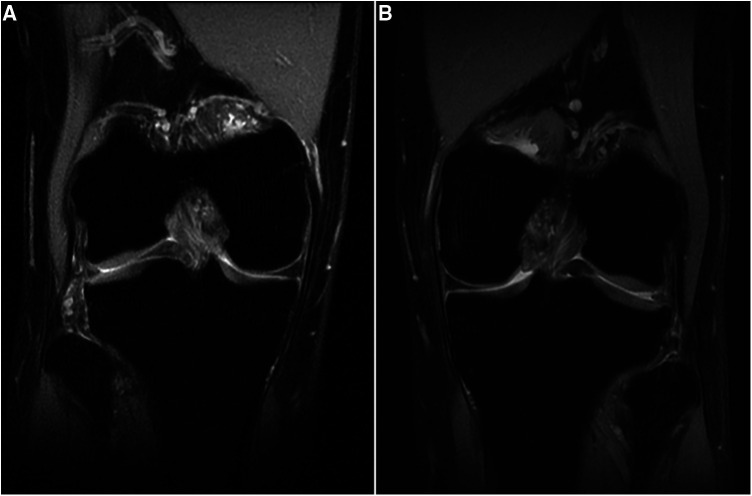
(**A**) Right knee and (**B**) left knee. Fat-suppressed proton density-weighted images of coronal views show bilateral thickening of lateral tibiofemoral cartilages with contour deformity of the subchondral bone beneath the lateral femoral condyle cartilage.

**Figure 2:**
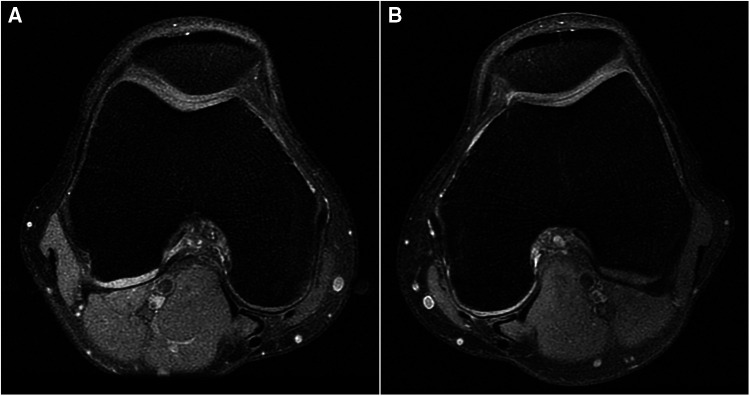
(**A**) Right knee and (**B**) left knee. Fat-suppressed proton density-weighted images of transverse views show that the posterior cartilages covered on both lateral femoral condyles also get thicker than the medial ones.

**Figure 3:**
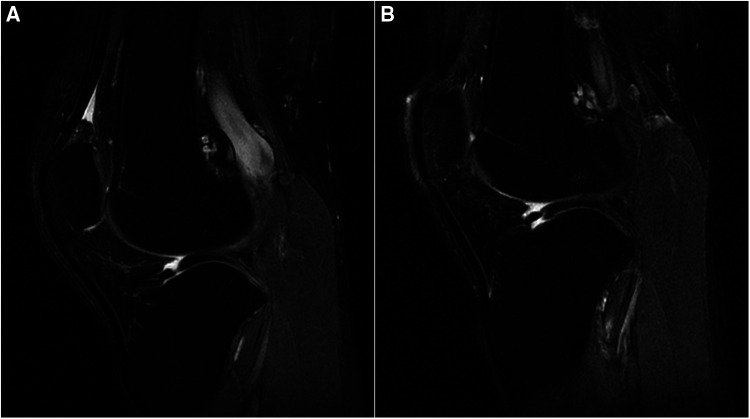
(**A**) Right knee and (**B**) left knee. Fat-suppressed proton density-weighted images of sagittal views show centrally displaced lateral menisci similar to the “central bow tie sign.”

**Figure 4:**
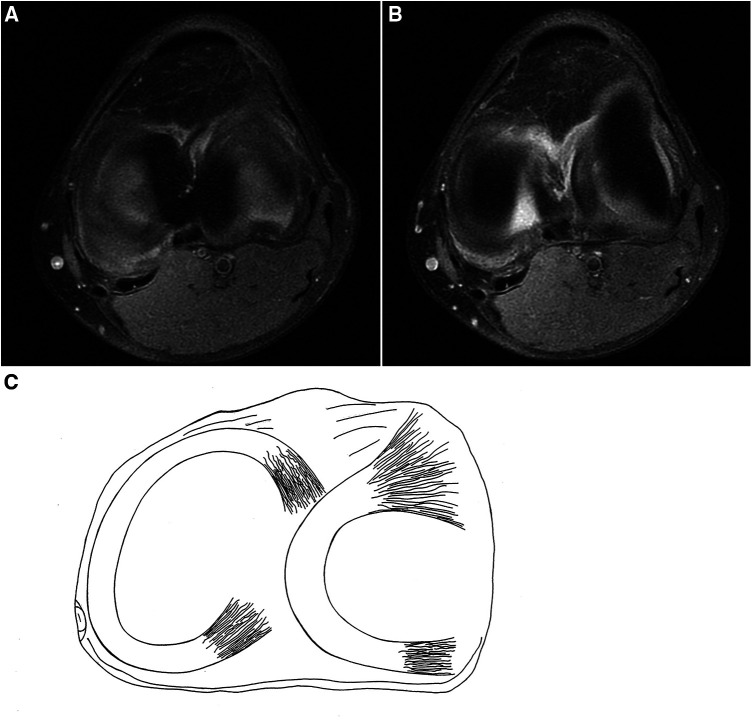
(**A,B**) Fat-suppressed proton density-weighted images of sagittal views of the left knee and (**C**) schematic drawing of the meniscal structure of the left knee. Almost whole of the lateral meniscus of the left knee dislocates to the center of the joint.

A 24-year-old male patient, 183 cm tall, experienced mild pain in both knees after continuously jogging for 20 min each day for 1 month. The pain was more pronounced in the left knee, especially during weight-bearing activities and while taking stairs, but it did not affect daily activities or cause knee locking. He reported no previous knee injuries or trauma. Physical examination revealed no swelling and increased temperature in either knee, and all tests for ligament abnormalities were negative. Both of his knees were examined using a 3-T MRI system (GE Discovery MR750) with a knee coil. MRI revealed slight effusion within the bilateral supra- and infrapatellar recesses. Fat-suppressed proton-weighted imaging revealed a significantly thickened lateral tibiofemoral cartilage with a smooth surface, without signs of wear and tear. The contour of the subchondral bone beneath the lateral femoral condyle cartilage was deformed, showing upward depression in the left femoral condyle and flattening in the right one (Figure 1). The anterior and posterior cartilages covering both lateral femoral condyles were also thicker than the medial ones (Figure 2). The normal intensity of the left linear and right triangular inferior horns of the bilateral lateral meniscus could be detected in the laterally sagittal views of the MRI. A band-shaped structure mimicking the “central bow tie sign” of both lateral menisci, presented as the connection of the anterior and posterior meniscal horns on two consecutive slices, was shown in the central joint sagittal view rather than in the lateral sagittal view (Figure 3). The lateral posterior joint spaces of both knees appeared narrow and vacuous. Almost all the left lateral meniscus (Figure 4) and most of the right lateral meniscus were dislocated to the central portion of the joint near the cruciate ligaments, with increased signal intensity consistent with degeneration within the meniscal structure. Based on the radiographic findings mentioned above, the young man was preliminarily diagnosed with thickened lateral articular cartilage complicated by one of the following conditions: BHT of both C-shaped lateral menisci, central tear of both lateral discoid menisci, or bilateral congenital RSM.

The patient stopped jogging and only maintained normal activities after the MRI examination. Invasive arthroscopy was not suggested by the orthopedist because there was no suspected knee locking and no persistent pain after relaxing for 1 month. The follow-up MRI after 1 year presented almost the same findings as before.

## Discussion

There are various morphological variants within the knee joint (3–5), including rare anatomic abnormalities of the menisci, such as discoid meniscus, RSM, double-layered meniscus, accessory meniscus, and hypoplastic meniscus (6). RSM is characterized by the presence of a bridge-like meniscal structure (7) between the two horns at the center portion of the joint, described as the so-called mirror image or reflection of the normal-appearing body of the meniscus (8), with a tapered morphology and smooth margins resembling a normal meniscus (9). RSM commonly exhibits a firm connection with surrounding soft tissues and can be disclosed during an arthroscopy procedure. An RSM is liable to be misinterpreted as a BHT of a normal C-shaped lateral meniscus or a central tear of a discoid meniscus, which displays a centrally mimicking meniscal structure, having an irregular and degenerative inner margin, lacking the tapered and sharp appearance (10). In coronal view, defects in the remainder of the meniscus in these two conditions could be easily observed. A BHT of the meniscus usually has a specific history of knee trauma, whereas a central tear of the lateral discoid meniscus appears to lack direct trauma. In most cases, both of these conditions usually require arthroscopic removal of the displaced redundant meniscal structure to relieve the knee pain and locking.

Congenital thickening of knee joint cartilage has rarely been reported before. Metatropic dysplasia, previously known as hyperplastic achondroplasia and commonly recognized in individuals of short stature, may present with thickened articular cartilage with a preservation of joint space, despite irregularities in the subchondral bone (1) involving bilateral knees. Previous literature on knee cartilage thickening has commonly focused on athletes with overused knees. Some studies have shown that repeated mechanical loading can thicken knee joint cartilage to adapt to the effects of high strain. Compared with sedentary controls, weightlifters typically show thickening in most of the measured sites of the medial and lateral condyles of the femur, regardless of whether these regions are weight-bearing or non-weight-bearing (11). Conversely, this case involves a mature adult who does not perform physical work. Furthermore, the fact that the thickened cartilage involved both the weight-bearing and non-weight-bearing regions of both lateral condyles of the femurs and the weight-bearing regions of both lateral tibial plateaus excludes the possibility of acquired thickening due to overuse. Studies involving twins have demonstrated that there was a large coefficient of variation in knee cartilage thickness within the control population, but the coefficient of the variation in knee cartilage thickness between twins was significantly reduced, which suggested a strong genetic influence on knee cartilage thickening (12). The thickened lateral articular cartilages, in this case, have created a narrow space that prevents the growth of the lateral meniscus. As a result, most of the meniscus structure had to move to the more spacious intercondylar notch area at the center of the joint during development, leading to the formation of such a rare aberration. This could be explained naturally, with both the dislocated meniscal structures being degenerative due to repeated contact with the cruciate ligaments, despite the young age of the patient. Recent jogging seemed to have triggered the symptoms, while the situation within the bilateral knee joints was supposed to have existed asymptomatically for multiple years. Consequently, after excluding diagnoses of congenital RSM, BHT of the C-shaped lateral meniscus, and central tear of the discoid meniscus, the displacement of the lateral meniscus to the intercondylar notch in the left knee of the patient was considered a consequence of congenital thickening of the lateral superior and inferior cartilages. Due to the similar appearance of thickened cartilages covering the right lateral tibiofemoral joint to those seen in the left knee, the right lateral meniscus exhibited features more similar to an RSM as the “central bow tie sign” could also be detected within the central portion of the right knee and a slimmer lateral meniscus structure could still be examined in its normal position. However, RSM, as one of the rare variants of the knee joint, has not been reported in conjugation with adjacent articular cartilage thickening in previous literature. Taken together, these findings provide further evidence to support the possibility that the thickening of lateral tibiofemoral cartilages in both knees can be attributed to congenital origin rather than a result of postnatal mechanical stimulation.

Both radiographic and clinical characteristics are summarized as follows. First, the “central bow tie sign” of the lateral meniscus body of both knees disappeared on lateral sagittal imaging. Instead, the bilateral anterior horns of the lateral menisci were flattened and the bilateral posterior horns were almost invisible between the superior and inferior thickened cartilages. Meanwhile, a triangular-shaped structure, similar to the lateral meniscus, was dislocated centrally to the intercondylar fossa, with clear margins and a hyperintense signal, without touching any of the articular surface. Second, the significant thickening of the lateral tibiofemoral cartilages and the loss of the downward convex shape of the corresponding lateral femoral condyles (being concave upward in the left knee and flattened in the right knee) suggested a congenital rather than postnatal deformation. Third, the patient, who was in his early adulthood, experienced knee pain during jogging without knee locking. The pain was more severe in the left knee, which was associated with the total displacement of the left lateral meniscus to the center of the joint. The deformation of the lateral meniscus of the right knee was more similar to that found in RSM. Fourth, the patient complained of mild knee pain, which could be relieved completely by avoiding sports activities without any clinical treatment.

With the development of spectral CT (13, 14) and quantitative MRI (15), more accurate assessment of the morphological measurement of the cartilage can be achieved, providing more helpful information for the diagnosis of cartilage lesions.

## Conclusion

As far as we know, this case seems to be the first report concerning the congenital thickening of lateral tibiofemoral cartilage and the subsequent dislocation of the lateral meniscus to the intercondylar notch, which might be a new variant in knee joint pathology. Although the displacing of the meniscal structure to the intercondylar notch can also be detected in BHT and RSM conditions, the unique morphological property of bilateral-involved thickening of lateral tibiofemoral cartilages could be helpful in explaining such meniscal alterations. The recognition of such radiographic findings could assist orthopedic surgeons in making the right diagnosis and avoiding invasive arthroscopic interventions in clinical practice.

## Data Availability

The original contributions presented in the study are included in the article/Supplementary Material, further inquiries can be directed to the corresponding author.
